# Diet-Related Changes of Short-Chain Fatty Acids in Blood and Feces in Obesity and Metabolic Syndrome

**DOI:** 10.3390/biology11111556

**Published:** 2022-10-24

**Authors:** Tamás Ilyés, Ciprian N. Silaghi, Alexandra M. Crăciun

**Affiliations:** Department of Molecular Sciences, University of Medicine and Pharmacy “Iuliu Hațieganu”, 400012 Cluj-Napoca, Romania

**Keywords:** short-chain fatty acids, diet, obesity, metabolic syndrome

## Abstract

**Simple Summary:**

Short-chain fatty acids are produced by the bacteria present in the large intestine. They are digestion products of fiber-containing foods and have many effects. Short-chain fatty acids appear to decrease weight gain and improve diseases related to obesity, with diet being the most important factor that modifies short-chain fatty acid levels in the body. The purpose of this article is to provide an overview and analysis of the variations of short-chain fatty acids in blood and stool in obesity-related conditions, thus helping to monitor these diseases.

**Abstract:**

Obesity-related illnesses are one of the leading causes of death worldwide. Metabolic syndrome has been associated with numerous health issues. Short-chain fatty acids (SCFAs) have been shown to have multiple effects throughout the body, both directly as well as through specific G protein-coupled receptors. The main SCFAs produced by the gut microbiota are acetate, propionate, and butyrate, which are absorbed in varying degrees from the large intestine, with some acting mainly locally and others systemically. Diet has the potential to influence the gut microbial composition, as well as the type and amount of SCFAs produced. High fiber-containing foods and supplements increase the production of SCFAs and SCFA-producing bacteria in the gut and have been shown to have bodyweight-lowering effects. Dietary supplements, which increase SCFA production, could open the way for novel approaches to weight loss interventions. The aim of this review is to analyze the variations of fecal and blood SCFAs in obesity and metabolic syndrome through a systematic search and analysis of existing literature.

## 1. Introduction

### 1.1. Short-Chain Fatty Acids

Short-chain fatty acids (SCFAs) are carboxylic acids with less than five carbon atoms [1]. These include methanolate, acetate (Ac), propionate (Prop), butyrate (Bu), and valerate; however, the three main SCFAs that have been extensively studied are Ac, Prop, and Bu. SCFAs are produced by the microbial fermentation of various dietary compounds, mainly fibers [2,3,4]. Ac, Prop, and Bu represent the majority of SCFAs produced in the colon [5,6].

Acetate is produced by metabolizing ethyl alcohol, by ingesting vinegar directly, or by the fermentation of dietary fiber [2,7]. The main area of synthesis is the cecum, followed by the ascending colon, with minimal production taking place in the distal colon [8]. Acetate is able to pass the blood–brain barrier and is found in varying concentrations in almost all tissues and liquids in the body [9,10]. Due to the fact that Ac is able to cross the blood–brain barrier, it has been shown to have a direct appetite-suppressing role in the central nervous system, more specifically in the hypothalamus [10]. Acetate also appears to be a ligand for the G protein-coupled receptors (GPR) 41 and GPR 43 [11,12].

Propionate is the second most abundant SCFA in the portal circulation after Ac [8]. Similarly to Ac, Prop has been shown to influence hunger and satiety; however, the exact mechanism has not yet been clarified [13]. It has been shown that Prop is the main ligand for GPR 41 and GPR 43, and in mice, Prop increases leptin (the main hormone involved in hunger and satiety regulation) levels, leading to a decrease in energy intake [14].

Butyrate is absorbed the least from the intestine, despite being produced in significant quantities. It acts mainly locally and is a main energy substrate for colonocytes [5,15]. Butyrate has also been shown, along with Prop, to regulate the division and proliferation of the large intestinal mucosa, with a possible role in preventing colon cancer [16,17], as well as other types of cancers [18].

GPR 41 and GPR 43, also known as free fatty acid receptors (FFAR) 3 and 2, respectively, have been shown to be present in significant quantities in adipocytes and colonocytes [19]. GPR 41 and 43 are G protein-coupled receptors, which were discovered earlier than their ligands, being initially called orphan G protein-coupled receptors, until Prop was demonstrated to be a ligand by Brown et al. [20] in 2003.

**Figure 1:**
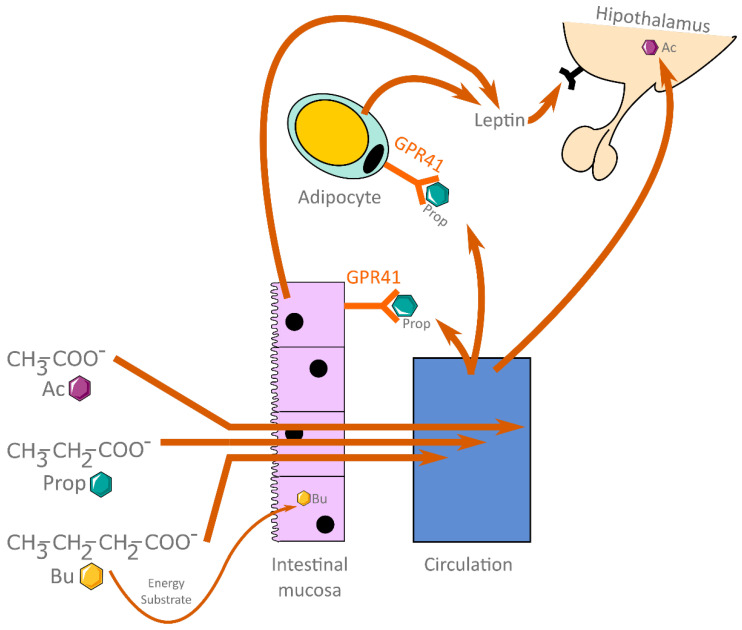
Summary of the key effects of the three main SCFAs. Abbreviations: Ac, acetate; Prop, propionate; Bu, butyrate; GRP, G protein-coupled receptor. The Figure was partly generated using Servier Medical Art, provided by Servier, licensed under a Creative Commons Attribution 3.0 unported license; https://smart.servier.com/ (accessed on 21 September 2022).

Figure 1 summarizes the key effects of the three main SCFAs (Ac, Prop, and Bu).

### 1.2. Obesity and Metabolic Syndrome

Metabolic syndrome is usually defined as a combination of hypertension, dyslipidemia, diabetes, and obesity [21]. Obesity in and of itself does not equal metabolic syndrome, but it is possible to have obesity without hypertension and diabetes. Metabolic syndrome, by increasing atherosclerosis and insulin resistance, increases the risk for stroke and myocardial infarction [22,23,24]. Increased blood glucose levels, independently of diabetes, were also found to be a risk factor for physical impairment [25].

Obesity is a major public health issue, especially in developed countries [26]. Increased body weight and obesity have been associated with multiple diseases that have a significant impact on quality of life and life expectancy [27]. The main causes of death globally are cardiovascular complications (such as myocardial infarction and stroke), as well as cancers of different origins, both being strongly associated with obesity [28,29].

Increased body weight is caused by two processes: excess energy intake and insufficient energy expenditure (due to decreased exercise). Increasing energy expenditure through exercise is the basis of obesity treatment and also of numerous chronic diseases, such as hypertension [30]. Only increasing exercise is not enough for an efficient weight loss, however, so a reduction in energy intake is also needed. There have been numerous diets developed along the years, but compliance to reducing the quantities of food is the most important factor in a successful reduction of energy intake [31].

Diet and exercise remain the predominant cornerstones in preventing and managing obesity and metabolic syndrome, as well as their inherent complications. The main source of SCFAs in the body is a result of microbial activity in the gut, especially the digestion of dietary fibers [2,3]. It is only natural that variations in diet will also influence SCFA production.

Previous reviews published about the effects of SCFAs on energy metabolism have either focused solely on diet [32] or on specific diseases related to obesity and metabolic syndrome [33,34,35]. The aim of this review is to provide an overview and analysis of the variations of SCFAs in feces and blood in obesity and metabolic syndrome and to identify novel ways to approach the monitoring of these diseases. The majority of studies analyzing SCFAs in obesity and metabolic syndrome included in this review were focused on diet and its influence on fecal and blood changes of SCFAs. To date, there have been no reviews to provide a combined and comprehensive overview of blood and fecal SCFA variations in both obesity and metabolic syndrome.

## 2. Methodology

### 2.1. Search Strategy

Databases accessible through the National Library of Medicine’s PubMed search engine were scrutinized for this review. Human, animal, and in vitro research papers were all selected. The search terms were constructed using the Medical Subject Headings (MeSH) function of PubMed. The following search terms were jointly selected by the authors: “(“Fatty Acids, Volatile”[Mesh]) AND “Obesity”[Mesh]” and “(“Fatty Acids, Volatile”[Mesh]) AND “Metabolic Syndrome”[Mesh]”. For the first search term, the search was carried out on the 30 June 2022 and, due to the large number of studies, was limited to the last 5 years, while for the second search term, the search was carried out on the 30 April 2022 and was limited to the last 10 years. This strategy yielded 291 and 86 results, respectively, for a total of 377 articles. Lists were made for each set of results which were cross-checked, and duplicates were eliminated. After the initial screening phase, based on the abstracts for each study, full-text articles were obtained, which were evaluated according to the inclusion and exclusion criteria.

### 2.2. Selection, Screening and Inclusion

The inclusion and exclusion criteria were agreed upon jointly by the authors. Only papers with abstracts were included in the initial screening phase. Human, animal, and in vitro studies were considered, and only articles written in English were included. Studies analyzing the effects of diet and SCFA supplementation were included.

Studies that did not measure total SCFA, Ac, Prop, or Bu levels and studies that lacked a clear description of materials and methods were excluded. Case reports and reviews were excluded as well.

**Figure 2:**
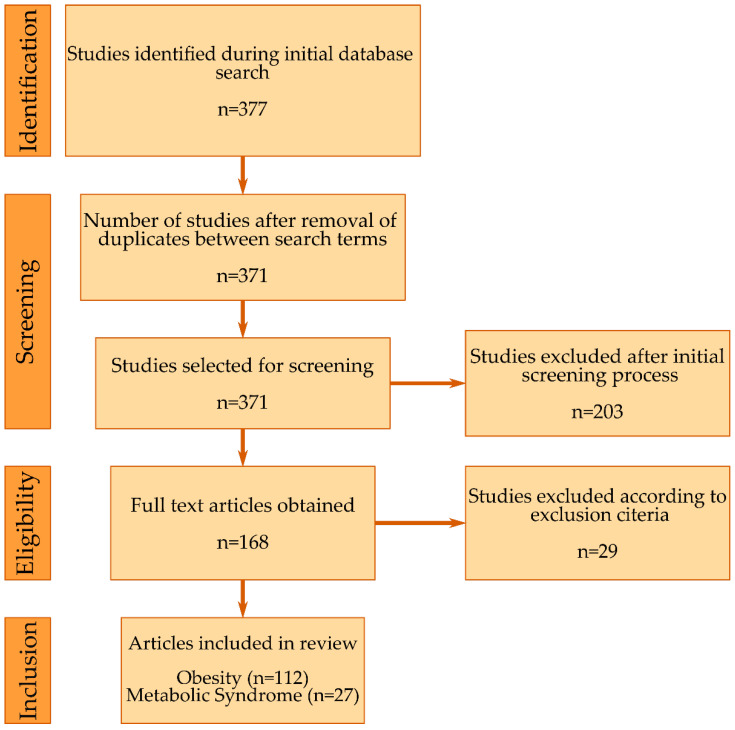
Flow diagram for the identification, selection, and inclusion process.

The study identification, selection, and inclusion process are summarized in Figure 2. After eliminating duplicates, in total, 139 articles were included in this review, 112 from the first search term containing obesity [36,37,38,39,40,41,42,43,44,45,46,47,48,49,50,51,52,53,54,55,56,57,58,59,60,61,62,63,64,65,66,67,68,69,70,71,72,73,74,75,76,77,78,79,80,81,82,83,84,85,86,87,88,89,90,91,92,93,94,95,96,97,98,99,100,101,102,103,104,105,106,107,108,109,110,111,112,113,114,115,116,117,118,119,120,121,122,123,124,125,126,127,128,129,130,131,132,133,134,135,136,137,138,139,140,141,142,143,144,145,146,147] and 27 from the second search term containing metabolic syndrome [148,149,150,151,152,153,154,155,156,157,158,159,160,161,162,163,164,165,166,167,168,169,170,171,172,173,174]. Of the total number of included articles, the majority of studies (n = 62) analyzed various parameters on high-fat-diet (HFD)-fed mice and rats.

## 3. Short-Chain Fatty Acids between Diet and Metabolism

### 3.1. Animal Studies

#### 3.1.1. Diet

Mice with metabolic syndrome have been shown to have lower levels of fecal short-chain fatty acids (fSCFA) [159], and obesity also reduced fecal butyrate (fBu) levels [104]. An HFD is a commonly used method of inducing diet-related obesity in animal test subjects. Mice on HFD were shown to have lower fecal acetate (fAc) [141] and blood acetate (bAc) [49] levels. There are even studies that have shown higher fSCFA levels in HFD-fed mice; this was probably a result of an increase in fiber intake and not a result of the HFD *per se* [89].

A Western diet also produced a reduction in fSCFA levels; however, this was ameliorated by subsequent exercise [41]. Exercise also increased fAC levels in the case of HFD-fed mice [106].

While there seems to be a connection between SCFA production and exercise levels, the exact nature of this interaction remains unknown. Until further studies analyze the effects of exercise on SCFA production, diet seems to be the main contributing factor to fecal and blood SCFA concentrations. Because HFD was used to induce obesity, the effects of various dietary supplements could be studied on an obese animal model. The effects of dietary supplements on SCFA levels of HFD-fed mice and rats are presented in Table 1.

In addition to HFD-fed mice, in the case of HFD-fed Göttingen Minipigs, whey increased fSCFA and fecal propionate (fProp) levels, and wheat bran increased fBu while decreasing fSCFA and fAc levels [80]. A less-used non-diet-induced obese mouse model is the ob/ob mutant (leptin deficient mice). Similarly to HFD mice, inulin (a dietary fiber from the fructans class) increased fSCFA in ob/ob mice as well [44].

In metabolic syndrome mice, caffeine and chlorogenic acid increased bAc, bProp, and bBu levels [156], while in the case of db/db mice (a type II diabetes and obese mutant mouse model), rutin (a citrus flavonoid) administration increased fSCFA [37]. Similarly, in monosodium glutamate (MSG)-induced obesity, fructo-oligosaccharides increased fAc, fProp, fBu, and bProp concentrations [99].

There was a clear trend in dietary supplements, especially fibers, increasing fecal and blood SCFA levels that were decreased as a result of HFD [56,60,65,66,68,70,72,73,77,81,85,86,103,105,110,111,112,113,114,118,120,122,124,126,130,136,137,138,139,144,158,160,162,167,169].

While an HFD-induced obese model can be used to analyze the effects of dietary supplements on obesity, the effects of dietary supplements are not exclusive to diet-induced obese animal models. Mice fed with a high-fructose diet showed an increase in fSCFA concentrations [161], and inulin-fed mice had increased fBu levels [39].

Even though these studies were carried out on animals that have not been on HFD, due to the close relationship between diet and obesity, the results showed a similar trend of increased SCFA levels after administration, especially fiber-containing dietary supplements [39,44]. This suggests that fiber-containing dietary supplements not only normalize and otherwise alter the SCFA profile due to HFD, but fibers also have the ability to increase SCFA production, regardless of the presence or absence of diet-induced obesity.

Another way of indirectly altering diet, especially in the case of obese subjects, is weight-reducing surgery, which produces a global decrease in food intake. Hypo-absorptive bariatric surgeries in obese rats resulted in higher levels of fProp and fBu, compared to sham surgery [46]. However, there are also conflicting results that show a reduction in fSCFA and blood short-chain fatty acid (bSCFA) levels in obese rats with Roux-en-Y gastric bypass, compared to sham surgery [74]. From the studies carried out so far, a global reduction on energy intake does not seem to have a clear effect on fecal and blood SCFA levels.

Prebiotics are nutrients that increase the concentration of certain bacteria in the gut that are considered beneficial to the host’s metabolism and health [175]. Prebiotics from acorn and sago combined with inulin increased fSCFA levels in HFD mice [119]. Commercial inulin-type fructans prebiotic administration in overweight beagles increased fSCFA levels as well [132]. As expected, prebiotics, especially ones that were fiber-related or that were co-administered with inulin, increased fSCFA levels [119,132].

#### 3.1.2. SCFA Supplementation

Short-chain fatty acids themselves can also be directly utilized as dietary supplements. Consequently, Table 2 contains the effects of SCFA administration on HFD-fed mice and rats. Furthermore, in the case of Western diet-fed mice, co-administration of inulin and Bu was shown to attenuate steatohepatitis [58].

There are studies conducted on non-diet-induced obese mice, in which Bu supplementation reduced body weight increase and reduced food intake [102]. Butyrate supplementation also reduced body weight gain and increased insulin receptor expression in obese Apo E knock-out mice [140]. Toll-like receptor 5 knock-out mice administered with Ac, Prop, and Bu had reduced food intake but increased total serum cholesterol and triglycerides [166].

**Table 2:** Effects of SCFA supplementation on HFD-fed mice and rats.

Acetate	Propionate	Butyrate
Ameliorated obesity [88]	Reduced body weight and fasting insulin levels [96]	Had no significant effect [82]
Normalized weight gain, insulin, TNF-α and leptin levels [48]	Increased adiponectin expression [135]	Altered gut microbiota to be similar to LFD [117]
Increased adiponectin expression [135]	Prevented weight gain [145]	Ameliorated obesity, steatohepatitis [38]
Prevented weight gain [145]		Reduced body weight gain, improved insulin response [92]
		Decreased leptin and insulin levels [146]
		Reversed HFD induced dysmetabolism [150]
		Increased adiponectin expression [135]

Abbreviations: TNF-α, tumor necrosis factor α; LFD, low-fat diet; HFD, high-fat diet.

Short-chain fatty acids consistently normalized or, in some cases, actually prevented HFD-induced alterations, such as weight gain [38,48,88,92,96,145], elevated insulin levels, and decreased leptin levels [48,92,96,146]. Consequently, SCFAs could be used in the future as a supplement to ameliorate weight gain.

#### 3.1.3. Probiotics and Gut Microbiota

Oral intake of *Bifidobacterium longum* and *Lactobacillus plantarum* increased fSCFA in obese, menopausal mice [61]. A similar increase in fSCFA levels was achieved as a result of gut microbiota transplantation from normal to leptin-deficient mice [108]. Contrary to the previous two studies on gut microbiota, antibiotic-induced microbial depletion significantly decreased fSCFA in otherwise healthy mice [133].

Considering the studies that involved probiotics, the majority focused on HFD-fed mice and rats. Thereby, Table 3 presents the effects of probiotics found in these studies.

Even though *Bifidobacterium* and *Clostridium* species are considered SCFA-producing bacteria [176], interestingly, their administration has not consistently produced increases in fSCFA [61,69], and in the case of *Clostridium*, actually decreased fSCFA [40]. Due to the complex and ever-changing nature of the gut microbiota and the numerous factors that influence its composition, it is difficult to correctly assess the ideal gut composition in any given host. The increase in one species, even if it is an SCFA-producing species, can lead to the decrease in other species at the same time, with global SCFA production suffering an imbalance as a result.

### 3.2. In-Vitro Studies

Using a colon model, it was shown that inulin and resistant starch increased Ac and Bu, but only in the lean model [45]. Similarly, prebiotic bread containing galacto-oligosaccharides increased Ac and Bu [168], and pumpkin skin fermentation produced high amounts of SCFAs [52]. Rice bran arabinoxylan increased total SCFA production in both normal weight and obese human gut microbiota [57]. Obese human gut microbiomes had higher production of Ac and Bu but lower production of Prop, compared to normal weight microbiomes; however, the total SCFA level was still higher in the obese microbiota [55]. In the case of gut microbiota from obese children, commercial prebiotics increased SCFA production; however, the magnitude of this increase was donor specific [94].

In an in vitro digestion model, propolis increased SCFA concentrations [42]. Bianchi et al. [131] showed, using the Simulator of the Human Intestinal Microbial Ecosystem, that citric pectin also increased Ac and Bu.

In precision-cut liver slices from metabolic-associated fatty liver disease from male mice, Bu treatment reduced fibrotic response but decreased fat oxidation [148]. Butyrate enhanced the immunomodulating effect of adipose-derived stem cells from obese and diabetic mice [67].

In vitro studies have the advantage of being able to precisely control all aspects of a given model, compared to animal subjects. The main drawback, however, is the fact that, by controlling all parameters perfectly, in vitro models might not be representative of the real-world conditions. Even though it is difficult to simulate obesity and metabolic syndrome in vitro, studies that assess the effects of nutrients and SCFAs on the gut microbiome still provide valuable insights into the function of this complex symbiosis.

### 3.3. Human Studies

Levels of fBu and fProp were found to increase progressively with body weight [84], and fSCFA was positively associated with the prevalence of obesity in a Japanese population [59]. Normal-weight individuals also had lower levels of fBu [71], and weight loss interventions lowered fSCFA levels [97]. Non-obese, non-alcoholic steatohepatitis patients with significant fibrosis had elevated fProp, compared to controls [90].

Obesity in pregnancy was associated with a reduction in fSCFA levels [62], while preeclampsia women had lower blood butyrate (bBu) and Bu-producing bacteria [75]. Exclusively breast-feeding mothers had higher milk Bu levels than mixed-feeding mothers, which was negatively associated with changes in infant weight [115].

In children, fAc, fProp, and fBu levels were positively correlated with BMI [51,78]. There were, however, conflicting results as well, which showed that obese children had lower fSCFA levels, compared to normal-weight children [128]. In obese adolescent girls, waist circumference was associated with fSCFA levels [101], while in obese adolescents, *Lactobacillus salivarius* administration did not alter fSCFA levels [172]. Post-bariatric surgery fAc, fProp, and fBu were significantly reduced and were positively correlated with BMI in obese adults [50].

Men with metabolic syndrome had significantly higher levels of bProp and bBu and lower levels of bAc, compared to healthy, lean men [157], and obese women had significantly higher fAc, fProp, and fBu compared to lean women [173]. Higher fProp was corelated with serum triglycerides, and fBu levels were also associated with metabolic syndrome [149].

Exercise increased fSCFA in lean but not obese subjects [142], behavioral weight loss and metformin treatment increased bAc, and metformin also increased bBu [64].

Butyrate administration reduced inflammation [121] and brain dopamine binding [151]. Levels of bBu were negatively corelated with distal neuropathic pain [91].

The abundance of gut *Firmicutes* bacteria belonging to the *Ruminococcaceae* family was positively associated with bAc levels [143], while the levels of *Bacteroidetes,* such as *Allistipes* and *Bacteroides,* were correlated with fProp levels [170]. The concentration of fSCFA was inversely associated with microbiome diversity [123]. Recipients of the metabolic syndrome donor fecal transplant had higher fProp and fBu but not fAc levels [154].

#### Dietary Supplementation

The effects of dietary supplements and various diets on SCFA levels in humans are summarized in Table 4.

Due to different dietary habits, a pertinent finding was that Ghanaians consumed more fiber and had higher fSCFA levels, compared to United States residents [127]. Levels of fBu were positively associated with starch intake and negatively with non-nutritive sweetener intake [107].

There are significantly fewer human studies compared to animal studies; however, the results of these generally coincide with the results of animal studies. (Fiber-rich diets increase fecal and blood SCAFs.) This suggests that the animal models (most frequently mice and rats) used to analyze the effects of diet on blood and fecal SCFA levels provide valuable information regarding the changes in SCFAs produced by diet.

### 3.4. Diet and SCFAs

Diet, unequivocally, has a significant influence on both fecal and blood levels of SCFAs, as well as the relative abundance of Ac, Prop, and Bu [36,37,39,41,42,43,44,45,47,52,54,56,57,60,63,65,66,68,70,72,73,76,77,80,81,83,85,86,89,93,95,98,99,100,103,105,110,111,112,113,114,118,120,122,124,125,126,129,130,131,134,136,137,138,139,144,147,149,152,153,155,156,158,160,161,162,163,164,165,167,168,169,171,174]. Diet can alter the SCFA profile both directly, through supplying a substrate for SCFA-producing bacteria, as well as indirectly, by altering the gut microbiome’s composition. HFD reduced SCFA concentrations by varying amounts in multiple studies [49,104,141], as did a Western-type diet [41]. Obesity and metabolic syndrome reduced SCFA levels as well [49,62,104,128,154,159]; however, there is not a clear trend, and obesity has been shown to increase SCFA concentrations in some populations [51,59,71,78,84,101]. Because the main result of HFD consumption is diet-induced obesity, it is not exactly clear if HFD reduced SCFA levels directly or if these alterations are actually due to the resulting obesity.

In terms of diet, supplementation with dietary fiber and/or high-fiber foods had the most profound effect on both fSCFA and bSCFA profiles, generally increasing SCFA concentrations [36,39,44,45,52,54,56,60,70,72,77,80,85,86,93,98,103,105,111,122,125,126,129,130,131,134,137,153,155,158,163,164,165,167,169,171]. This, however, should not come as a surprise, given the fact that the main source of SCFAs is the bacterial fermentation of fibers and resistant sugars [176].

It is of note, however, that there are multiple articles demonstrating that fats increased SCFA production as well [124,174]. This suggests that there might be an alternate mechanism present in the gut microbiota that enables the production of SCFAs from sources other than indigestible fibers.

The effects of fiber supplementation have been extensively studied, but studies on other types of dietary supplements (mainly proteins and lipids) are lacking. Even though it is clear that fibers increase the production of SCFAs, there are multiple studies that have shown this effect in the case of other types of dietary supplements as well [118,124,174]. Further studies should focus on evaluating the effects of other supplements in modifying SCFA production.

**Figure 3:**
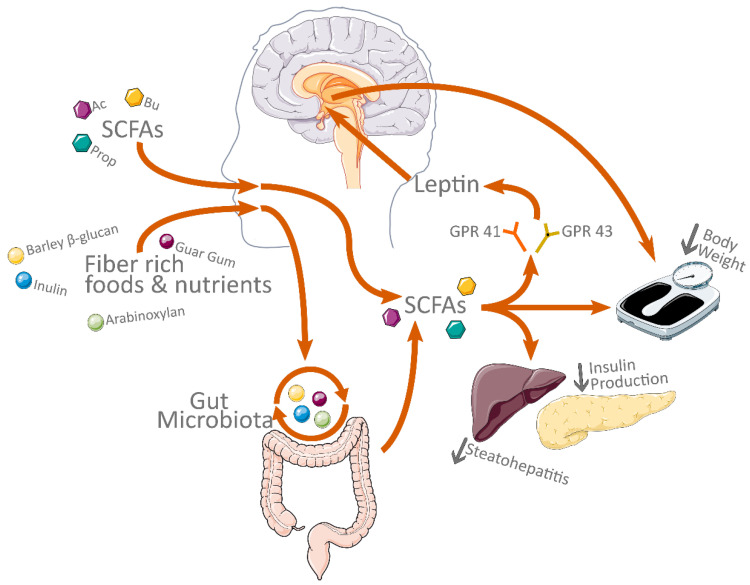
Overview of the effects of dietary supplements and SCFAs. Abbreviations: Ac, acetate; Prop, propionate; Bu, butyrate. The Figure was partly generated using Servier Medical Art, provided by Servier, licensed under a Creative Commons Attribution 3.0 unported license; https://smart.servier.com/ (accessed on 21 September 2022).

Direct supplementation of SCFAs, as well as increases resulting from diet, produces a host of beneficial effects from weight loss to normalization of insulin levels and amelioration of hepatic steatosis (Figure 3).

### 3.5. Gut Microbiota and SCFAs

Even though the main focus of this review was not the influence of probiotics on the gut microbiota composition and functionality, there are studies that have also analyzed this perspective.

There are many microbes present in the human gut that have been shown to produce SCFAs [176]. Supplementation with probiotics containing such microbes has not produced a consistent increase in SCFA production, in some cases, had the exact opposite effect of reducing fSCFA levels [40,69]. Thus, a study demonstrated that the concentration of fSCFAs was inversely associated with microbiome diversity [123].

This would suggest two possibilities. The first one is that the substrate supplied to the gut microbes actually has a more important role in determining the production and relative abundance of SCFAs. This hypothesis is supported by the fact that, in some conditions, known SCFA-producing bacteria increased fSCFA levels as expected [53,79,87,116], while in others, it actually decreased SCFA production [40,69]. The key to influencing SCFA production might not be artificially increasing the abundance of a certain species of bacteria through probiotics but of increasing the available substrate. This would create an environmental selection pressure that would favor multiple SCFA-producing bacteria on one hand, and on the other hand, increase the production of SCFAs by default, due to a higher substrate supply.

The other possibility is exactly the opposite of the first one, which is that there is actually one or just a small number of species of SCFA-producing bacteria that are actually responsible for the majority of the produced SCFAs. This hypothesis is somewhat supported by the fact that fSCFA levels were inversely associated with gut microbiota diversity [123]. Research that would prove or disprove this hypothesis would need to analyze the effects of various probiotic supplements in modifying the SCFA profile under the same conditions and preferably in the same individuals, both in animals and humans.

## 4. Conclusions

This is the first review to encompass studies related to diet that analyze both fecal and blood SCFA levels in obesity and metabolic syndrome. The effects of the gut microbiota, while being widespread, are sparsely understood. SCFAs are only a small part of the wide range of metabolites produced by gut bacteria, which have a profound effect on their host’s metabolism and homeostasis.

It is clear that both the gut microbiota functionality, as well as its composition, are closely related to diet, and that dietary fibers increase the production of SCFAs. The exact mechanism by which gut microbiota composition affects this relationship remains unclear.

While it has been shown that increasing fecal and blood SCFA levels lead to numerous beneficial results, especially in the case of obesity, the mechanism by which SCFAs produce these changes is a matter of debate. Short-chain fatty acids, mainly Prop, are known ligands of GPR 41 and GRP 43, through which they have been shown to increase leptin expression. Leptin is responsible for decreasing energy intake through its action on the hunger centers of the central nervous system. This is supported by multiple studies demonstrating that increased SCFA levels decreases weight gain; however, this is only one hypothesis for the mechanism of action of SCFAs. This review envelops the latest research caried out on the effect of diet and dietary supplements on the fecal and blood levels of SCFAs, as well as an overview of the SCFA-mediated effects of the gut microbiota. Diet influences the host microbiome and has a profound effect on energy metabolism regulation. Uncovering the exact nature of this host–microbiota interplay could yield novel ways to approach the monitoring of energy metabolism and treatment of obesity-related conditions, as well as establishing an optimal diet to aid in the treatment of obesity.

## Figures and Tables

**Table 1:** Effects on SCFA levels of dietary supplements on HFD-fed mice and rats.

Dietary Supplement	SCFA Variations	Reference
Bilberry	↑fSCFA, bSCFA	[111]
Guar gum	↑fAc, fBu	[56]
	↑fSCFA	[167,169]
Erythritol	↑fSCFA, bSCFA	[60]
Nobiletin	↑fSCFA	[66]
Baicalin	↑fSCFA	[112]
Barley β-glucan	↑fSCFA	[70]
	↑fAc, fProp	[72]
	↑fBu	[137]
Betaine	↑fAc, fBu	[73]
Coarse cereal mixture	↑fSCFA	[77]
Phlorizin	↑fSCFA	[81]
Inulin	↑fSCFA	[85]
Pinto beans	↑fBu	[86]
*Deinococcus geotermalis* modified chestnut starch	↑fAc	[103]
Green banana	↑fSCFA	[105]
β-hydroxy-β-methylbutyrate	↑fProp	[113]
Tea extract	↑fSCFA	[114]
Chondroitin sulfate	↑bSCFA	[118]
Jamun fruit extract	↑fSCFA	[120]
Euglena + vegetables	↑fSCFA	[122]
Flaxseed fiber	↑fSCFA	[126]
Bacterial cellulose + konjac glucomannan	↑fAc, fProp, fBu	[130]
Hydroxysafflor yellow A	↑fSCFA	[136]
Anthocyanins	↑fBu	[139,144]
Bletilla striata	↑fAc ↓fProp	[152]
Cranberry extract + isomalto-oligosaccharides	↑fSCFA, fBu	[160]
Chickpea α-galacto-oligosaccharides	↑fProp, fBu	[162]
Caffeine + epigallocatechin-3-gallate	↑fAc, fProp	[65]
Trilobatin	↑fProp, fBu	[68]
Xiexin Tang	↑fSCFA	[110]
Gamma-aminobutyric acid enriched rice bran	↑fProp, fBu	[158]
Trans-10,cis-12 Conjugated linoleic acid	↑bAc, fBu	[138]
Lard fat + sucrose	↑bProp	[124]
Lard fat + sucrose + fructose	↓bAc, bBu	[124]

Abbreviations: ↑, increase; ↓, decrease; fSCFA, fecal short-chain fatty acids; bSCFA, blood short-chain fatty acids; N/A, not applicable; fAc, fecal acetate; fBu, fecal butyrate; fProp, fecal propionate; bAc, blood acetate.

**Table 3:** Effects of probiotics on HFD-fed mice and rats.

Probiotic	SCFA Variations	Reference
*Bifidobacterium adolescentis*	↓fSCFA	[69]
*Clostridium cochlearum*	↓fSCFA	[40]
*Eurotium cristatum*	↑fBu	[109]
*Lactobacillus plantarum*	↑fSCFA	[87]
*Lactobacillus reuteri*	↑fBu, bBu	[53]
*Lactobacillus sakei*	↑fSCFA, bSCFA	[116]
	↑fBu	[79]

Abbreviations: ↑, increase; ↓, decrease; fSCFA, fecal short-chain fatty acids; fBu, fecal butyrate; bBu, blood butyrate; bSCFA, blood short-chain fatty acids.

**Table 4:** Effects of dietary supplements and various diets on SCFA levels in humans.

Dietary Supplement	SCFA Variations	Reference
Arabinoxylan	↑bAc, bBu	[171]
	↑fAc, fBu	[163]
	↑fProp	[93]
Inulin	↑bAc	[134]
Grape pomace	↓fBu	[83]
Rye	↑bBu	[36]
Yacon flour	↓fSCFA	[63]
Energy-restricted diet	No effect on bSCFA	[100]
	↓fAc, fBu	[43]
Intermittent-fasting diet	No effect on bSCFA	[100]
Mediterranean diet	↑bBu	[95]
Vegan diet	↑fBu	[47]
Barley β-glucan	↑fProp	[155]
Galacto-oligosaccharides	No effect on bSCFA or fSCFA	[147]
Almond	No effect on fSCFA	[76]
Pea fiber	↑fAc	[125]
Whole-grain cereal	↑bAc, bSCFA	[153]
	↑bProp	[165]
Wheat bran	↑bAc, bProp, bSCFA ^1^	[54]
Refined cereal	↑bAc, bSCFA	[153]
Mango	↑fBu ^2^	[129]
Resistant starch type 4	↑fProp, fBu	[164]
Juçara berry	↑fAc	[98]
Saturated fat	↑fSCFA	[174]
Monounsaturated fat	↑fSCFA	[174]

^1^ only in obese individuals; ^2^ only in lean individuals. Abbreviations: ↑, increase; ↓, decrease; bAc, blood acetate; bBu, blood butyrate; fAc, fecal acetate; fBu, faecal butyrate; fProp, fecal propionate; fSCFA, fecal short-chain fatty acids; bSCFA, blood short-chain fatty acids; bProp, blood propionate.

## Data Availability

Not applicable.
